# Early and mid-term outcome in terms of functional and hemodynamic performance of the st. Jude regent 19-mm aortic mechanical prosthesis versus 19-mm carpentier edwards aortic biological prosthesis

**DOI:** 10.1186/s13019-015-0361-3

**Published:** 2015-11-06

**Authors:** Edvin Prifti, Massimo Bonacchi, Fadil Ademaj, Gabriele Giunti, Giampiero Esposito, Arben Baboci, Gani Bajraktari, Altin Veshti, Aurel Demiraj, Vittorio Vanini

**Affiliations:** 1 Division of Cardiac Surgery, University Hospital Center of Tirana, Tirana, Albania; 2 Division of Cardac Surgery, Humanitas Gavazzeni Clinic, Bergamo, Italy; 3 Department of Cardiovascular Sciences, Policlinico Careggy, University of Florence, Florence, Italy; 4 University Hospital Center of Pristina, Pristina, Kosovo

**Keywords:** St Jude Medical Regent 19-mm mechanical aortic prothesis, Carpentier-Edwards bioprothesis, Indexed effective orficice area, Indexed left ventricular mass

## Abstract

**Background:**

The aim of the present study is to compare the early and mid-term clinical and hemodynamic results of the aortic valve replacement (AVR) with a St Jude Medical Regent 19-mm prosthesis (SJMR-19) versus Carpentied-Edwars bovine pericardial 19-mm valve (CE-19).

**Methods:**

Between January 2002 and January 2012, 265 patients (Group I) and 58 patients (Group II) with underwent AVR with a SJMR-19 and CE-19 respectively. There were no significant differences between groups regarding the demographic and preoperative echocardiographic data. Thirty-six patients in Group I and 4 in Group II required annulus enlargement in association or not with septal myectomy. The mean follow-up was 34 ± 18.5 months (range 5–60 months).

**Results:**

There were 14 (5.3 %) hospital deaths in Group I versus 4 (6.8 %) in Group II (*p* = 0.86). The multivariate logistic regression analysis identified the LVEF ≤ 35 % (*p* = 0.001), combined operation (*p* = 0.0005), CPB (*p* = 0.033), age (*p* = 0.011), annulus enlargement (*p* = 0.0009), reoperation (*p* = 0.039) and chronic renal failure (*p* = 0.011) as strong predictors for early postoperative death. Within 1 year after surgery peak pulmonary artery pressure, interventricular septal and left ventricular posterior wall thickness decreased significantly in both groups. The M-TPG was 15.7 ± 6.5 mmHg in Group I versus 17 ± 7 mmHg in Group II (*p* = 0.19). The multivariate regression analysis revealed the annulus enlargement (*p* = 0.018), small EOA_i_ (*p* = 0.00004), postoperative LVM_i_ (*p* = 0.0001) and BSA (*p* = 0.019) as strong predictors for higher M-TPG. The postoperative LVM_i_ was 119 ± 22.5 gm/m^2^ in Group I and 122 ± 22 gm/m^2^ in Group II (*p* = 0.37), significantly lower than the respective preoperative values 162.5 ± 34 gm/m^2^ (Group I) and 168 ± 30 gm/m^2^ (Group II). The actuarial survival and cumulative free-reoperation actuarial survival at 5 years follow-up were 96.7 and 94.5 % respectively in Group I and 97 and 91 % in Group II.. There were non significant differences between groups regarding the actuarial survival and cumulative free-reoperation survival. The Cox model identified the older age (*p* = 0.022), LVEF ≤ 35 % (*p* = 0.009), reoperation (*p* = 0.018), combined surgery (*p* = 0.00075) and annulus enlargement (*p* = 0.033) as strong predictors for poor actuarial free-reoperation survival.

**Conclusions:**

Both the SJMR-19 and CE-19 offers excellent postoperative clinical and hemodynamic outcome in patients with small aortic annulus. The LV hypertrophy and transvalvular gradients are reduced significantly indenpendently of the employed SJMR-19 or CE-19 prosthesis. Our data support recent suggestions that small valve size does not influence intermediate free-reoperation survival. The CE-19 is an excellent alternative to SJMR-19 in old patients.

## Background

Aortic valve replacement (AVR) in patients with a small aortic annulus is often challenging for the surgeon in terms of prosthesis selection. The goal of surgery is to replace the valve with a competent, nonstenotic prosthesis that allows resolution of patient symptoms and normalization of hemodynamics. Although it is easy to define a competent prosthesis, defining what constitutes a nonstenotic prosthesis has been a challenge. AVR with a small prosthetic valve is technically straightforward and commonly performed, but it may result in a patient-prosthetic mismatch resulting in a high residual outflow gradient, the significance of which remains the subject of controversy [1–8]. Annular enlargement allows for insertion of a larger aortic prosthesis, but it too may introduce increased surgical risks [9]. Use of stentless valves or homografts results in lower residual postoperative gradients, but implant procedures are technically more demanding, leading to increased total ischemic time [10, 11] and the log-term durability remain unknown [11, 12]. The Carpentier-Edwards (CE) pericardial valve, a stented tissue valve made of bovine pericardium, is widely used in patients with small aortic roots. Rao et al. [10] provided evidence that stentless and stented valves have similar hemodynamic profiles in the small aortic root when matched on true measured internal diameters demonstrating that the clinical benefit of the stentless porcine valve may be due to patient selection or the lack of a rigid stent in the small aortic root, but it is not due to hemodynamic superiority over stented aortic valves of similar sizes.

Bileaflet mechanical valves are still the most implanted cardiac valve substitutes in the aortic position. Their excellent durability and low incidence of cardiac-related complications have been widely reported [3, 13]. The St. Jude Medical Regent 19-mm mechanical aortic prosthesis (SJMR-19) valve is the next-generation bileaflet mechanical prosthetic aortic valve, constructed of pyrolytic carbon which has a modified external profile that achieves a larger geometric orifice area without changing the existing design of the pivot mechanism or blood-contact surface areas.

The aim of the present study is to report the early and mid-term clinical and hemodynamic results of the AVR with a SJMR-19 versus CE-19.

## Methods

Between January 2002 and January 2012, 265 patients (Group I) and 58 patients (Group II) with aortic valve disease underwent AVR with a SJMR-19 and CE-19 respectively. Valve replacement was performed for rheumatic, congenital or degenerative valve disease and severe aortic valve stenosis with or without regurgitation. The investigational board (The IRB of the division of cardiac surgery at the University Hospital Center of Tirana, Albania, the scientific committee of the division of cardiac surgery of Bergamo, Italy, and the IRB of the division of experimental surgery, cardiac surgery section, University of Florence, Italy) approved the study protocol as a multi-institutional study and written informed consent was obtained from all patients. The prosthesis type was basically decided by the surgeons’ preference in selected cases, patients’ decision regarding the long-term anticoagulation and older age.

### Patients’ characteristics

**Fig. 1:**
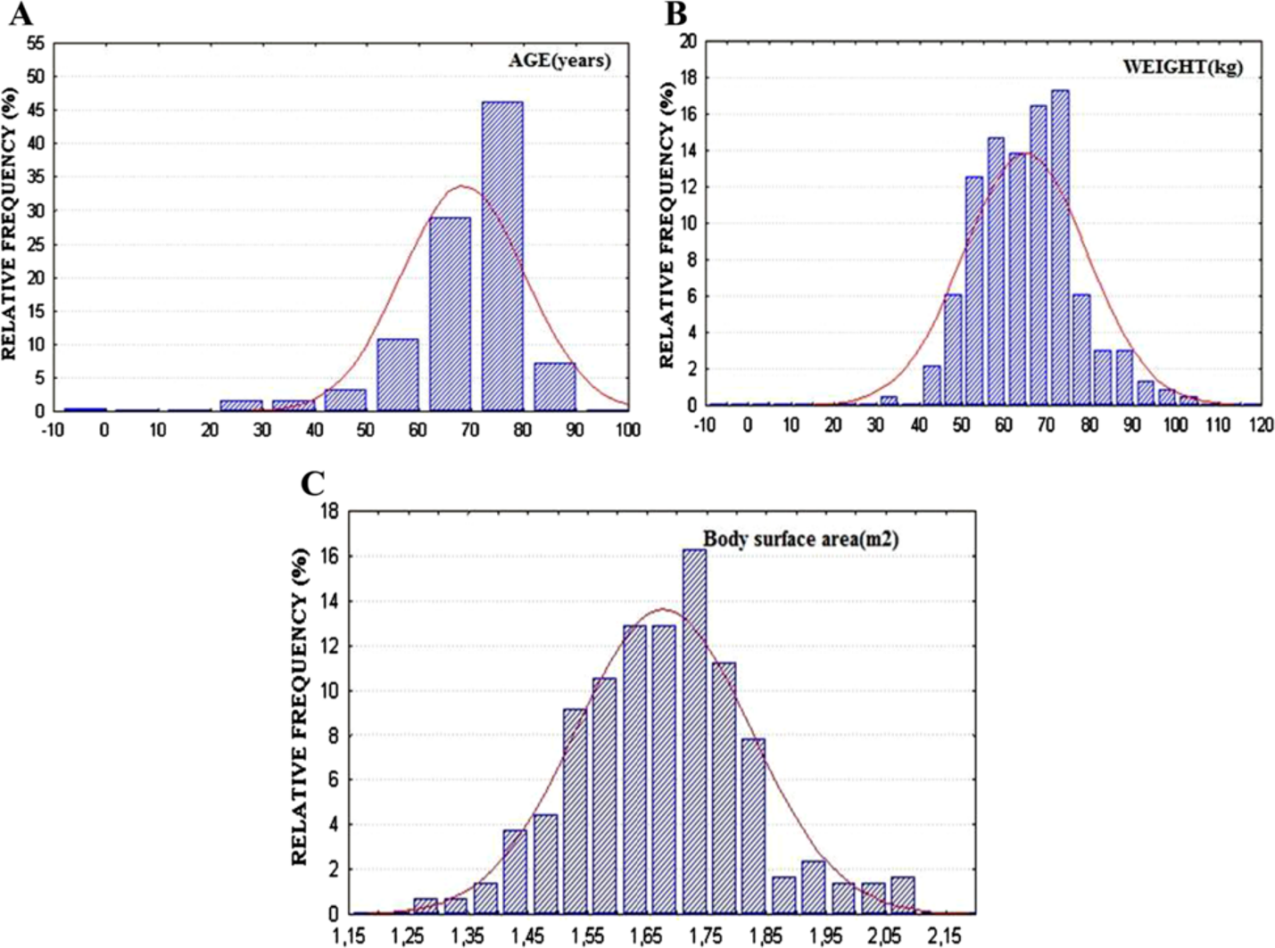
Distribution of **a** age; **b** weight and **c** body surface area

Preoperative demographic data are given in Table 1. Mean age was 67.5 ± 12.72 years in Group I and 71 ± 16 years in Group II (*p* = 0.07). Almost 102 patients from Group I were older than 65 years. In this subset of patients a mechanical valve SJMR-19 was employed due to technical difficulties of employing a biological valve because of the very small aortic annulus. In some other cases was the patient’s preference or not availability of the CE-19. The age, weight and BSA distribution for all patients are given in Fig. 1a-c. Preoperative echocardiographic data are given in Table 2. There were no significant differences between groups regarding the demographic and preoperative data.

**Table 1:** Demographic data

Variables	Group I	Group II	*p*
	(*n* = 265)	(*n* = 58)	
Males	1 (19.3 %)	14 (24 %)	0.51
Mean age (years)	67.5 ± 12.72	71 ± 16	0.07
Mean Canadian Cardiovascular Class	1.41 ± 0.62	1.34 ± 0.63	0.44
Mean New York Heart Association class	2.4 ± 0.75	2.34 ± 0.82	0.6
Mean height (cm)	159.7 ± 7.7	161.8 ± 12	0.1
Mean weight (kg)	66.2 ± 12.7	67 ± 13	0.67
Mean Body Surface Area (m^2^)	1.67 ± 0.14	1.69 ± 0.25	0.41
Mean Body mass Index (kg/m^2^)	26 ± 4.8	25.5 ± 4.4	0.47
Aortic stenosis	223 (84 %)	48 (82.7 %)	0.95
Diabetes	35 (13.2 %)	6 (10.4 %)	0.71
Insulin therapy	7 (2.7 %)	1 (1.7 %)	0.95
Hypercholesterolemia	82 (31 %)	16 (27.6 %)	0.87
Hypertension	118 (44.5 %)	21 (36 %)	0.31
Hypothyroidism	15 (5.7 %)	1 (1.7 %)	0.36
Smoking history	61 (23 %)	10 (17.3 %)	0.43
Chronic renal failure	11 (4.2 %)	6 (10.4 %)	0.11
Cerebrovascular disease	4 (1.5 %)	3 (5.2 %)	0.22
Previous transitory ischemic arrest	17 (6.4 %)	6 (10.4 %)	0.44
Previous carotid endarterectomy	7(2.7 %)	0	0.45
Previous peptic ulcer	21 (8 %)	5 (8.6 %)	0.93
Peripheric vascular disease	11 (4.2 %)	2 (3.5 %)	0.90
Preoperative arrhythmias	24 (9 %)	5 (8.6 %)	0.88
Permanent pacemaker	7 (2.7 %)	2 (3.5 %)	0.92
Nonelective	10 (3.8 %)	6 (10.4 %)	0.08
Endocarditis	11 (4.2 %)	4 (7 %)	0.58
Reoperation	31 (11.7 %)	4 (7 %)	0.41
III-IV reoperation	7 (2.7 %)	0	0.45
Ischemic heart disease	37 (14 %)	7 (12 %)	0.86
Myocardial Infarction	12 (4.5 %)	3 (5.2 %)	0.89
Chronic obstructive pulmonary Disease	31 (11.7 %)	8 (13.8 %)	0.83
Congestive heart failure	44 (16.6 %)	9 (15.5 %)	0.9
Left Ventricular Ejection Fraction ≤ 35 %	33 (12.5 %)	6 (10.4 %)	0.82
Mean Euroscore	6.9 ± 2	7.1 ± 2.9	0.51

**Table 2:** Preoperative echocardiographic data between groups

Variables	Group I	Group II	*p*-value
	(*n* = 251)	(*n* = 54)	
Diameter of the ascending aorta (mm)	29.3 ± 4.8	28.8 ± 5.3	0.5
Diameter of the left atrium (mm)	47 ± 10.5	45 ± 5	0.17
End-diastolic right ventricle diameter (mm)	22.3 ± 7.2	22.7 ± 6.8	0.71
End-diastolic LV diameter (mm)	48 ± 7.2	47.8 ± 7.6	0.86
End-systolic LV diameter (mm)	27.3 ± 7.6	28.3 ± 6.3	0.37
End-diastolic IVS thickness (mm)	13.3 ± 2.5	13.5 ± 3	0.61
End-systolic IVS thickness (mm)	17.8 ± 3.5	18 ± 5.4	0.73
End-diastolic LV PW thickness (mm)	11.2 ± 1.7	11.6 ± 2.5	0.16
End-systolic LV PW thickness (mm)	17.5 ± 3.5	17.7 ± 3.8	0.71
Shortening Fraction (%)	43.17 ± 8.6	45 ± 11	0.18
End-diastolic LV volume (ml)	101.5 ± 34.2	105 ± 31	0.49
End-systolic LV volume (ml)	44.2 ± 25	46 ± 20	0.62
Indexed end-diastolic LV volume (ml/mq)	60.4 ± 20	62 ± 23	0.61
Indexed end-systolic LV volume (ml/mq)	26.6 ± 15	27 ± 11	9.85
Left ventricular ejection fraction (%)	56.8 ± 12.6	54 ± 17	0.17
VmaxE (cm/s)	112 ± 58	120 ± 52	0.35
VmaxA (cm/s)	124 ± 41.5	132 ± 39	0.2
Mitral valve regurgitation grade	2.13 ± 0.8	1.97 ± 0.9	0.19
Peak pulmonary artery pressure (mmHg)	49.3 ± 14.7^a^	48 ± 12^a^	0.55
Left ventricular mass (gm)	271 ± 58	285 ± 60	0.11
Left ventricular mass index (gm/m^2^)	162.5 ± 34	168 ± 30	0.27
Peak transvalvular gradient (mmHg)	102.4 ± 24	108 ± 23	0.12
Mean transvalvular gradient (mmHg)	61 ± 16	60 ± 10	0.66
Aortic valve regurgitation grade	2.1 ± 0.8	2 ± 0.6	0.39
Peak trans tricuspid valve gradient	39.6 ± 12	37.5 ± 14	0.26
R/spess	2.13 ± 0.5	2.2 ± 0.7	0.39
R/spessmed	2.16 ± 1.16	2 ± 1.1	0.35

*LV* left ventricle, *IVS* Interventricular septum, *PW* posterior wall

### Anaesthesia and cardiopulmonary bypass

The inducement of anaesthesia consisted in: Fentanyl (25–30 γ/Kg), Diazepam (0,2 mg/Kg) and Bromure of Pancuronium (0,1 mg/Kg), and maintained with Ramifentanil (1–3 γ/Kg/min), Propofol and Isofluorane if necessary. The right atrium was cannulated using a double stage cannula. Normothermia and intermittent anterograde warm blood cardioplegia was employed. The left ventricle was vented through the right superior pulmonary vein.

### Operative techniques

The aortic annulus was debrided meticulously and measured with a snugly fitting sizer. Prosthesis size was selected according to the size of the aortic annulus. The AVR with a larger size aortic prosthesis such as 21-mm valves was tempted in all cases. AVR prosthesis size and type (Carpentier-Edwards bovine pericardial or SJMR were left to the discretion of the operating surgeon. We rim the annulus with interrupted 2–0 Ethibond (Ethicon, Somerville, NJ) mattress sutures, with or without Teflon (Impra Inc, a subsidiary of L.R. Bard, Tempe, AZ) pledgets. The prosthesis were inplanted in the supraannular position in all cases. Thirty-six patients in Group I and 4 in Group II required annulus enlargement in association or not with septal myectomy. We enlarge the aortic annulus by extending the standard oblique aortotomy down through the commissure between the left and noncoronary sinuses, about 1 to 1.5 cm into the base of the anterior mitral leaflet and a long elliptical Hemashild patch was employed to enlarge the left ventricular outflow tract and to close the entire aortotomy. The operative and early postoperative data are given in Table 3.

**Table 3:** Intraoperative and early postoperative data

Variables	Group I (%)	Group II (%)	*p*-value
Mean cardiopulmonary bypass time (minutes)	104 ± 41	98 ± 53	0.34
Mean aortic cross-clamping time (minutes)	82 ± 32	78 ± 37	0.41
Annulus enlargement	36 (13.6 %)	4 (6.9 %)	0.24
Mitral valve replacement	31 (11.7 %)	4 (6.9 %)	0.41
Coronary artery bypass grafting	37 (14 %)	7 (12 %)	0.87
Total grafts	66 (25 %)	12 (20.7 %)	
Associated carotid endarterectomy	4 (1.5 %)	0	0.78
Tricuspid valve repair	8 (3 %)	4 (6.9 %)	0.30
Postoperative outcome			
Mean mechanical ventilation (hours)	9.8 ± 9.5	12.3 ± 14	0.10
Mean Intensive Care Unit stay (days)	3 ± 6.4	2.7 ± 2.6	0.73
Readmitted in Intensive Care Unit	8 (3 %)	3 (5.2 %)	0.68
Postoperative complications	52 (19.6 %)	14 (24 %)	0.55
Low cardiac output	39 (14.7 %)	9 (15.5 %)	0.96
Myocardial infarction	10 (3.8 %)	2 (3.5 %)	0.79
Ventricular arrhythmias	27 (10 %)	5 (8.6 %)	0.91
Heart Block	4 (1.5 %)	1 (1.7 %)	0.64
Reoperation for bleeding	15 (5.7 %)	3 (5.2 %)	0.87
Blood used	126 (47.6 %)	30 (52 %)	0.67
Pulmonary complications	17 (6.4 %)	5(8.6 %)	0.75
ARDS	3 (1.1 %)	1 (1.7 %)	0.78
Pneumonia	9 (3.4 %)	2 (3.5 %)	0.71
Reintubation	12 (4.5 %)	3 (5.2 %)	0.90
Neurological complications	9 (3.4 %)	0.33	
Stroke	2 (0.75 %)	0	0.80
Infectious complications	14 (5.3 %)	4 (6.9 %)	0.87
Septicemia	5 (2 %)	1 (1.7 %)	0.65
Deep sternal wound infection	4 (1.5 %)	2 (3.5 %)	0.64
Superficial sternal wound infection	5 (2 %)	1 (1.7 %)	0.65
Renal complications	19 (7.2 %)	9 (15.5 %)	0.08
Hemodialysis	7 (2.6 %)	3 (5.2 %)	0.56
Multi organ failure	5 (2 %)	1 (1.7 %)	0.65
Coagulopathy	5 (2 %)	1 (1.7 %)	0.65
Hospital death	14 (5.3 %)	4 (6.9 %)	0.87
Hospital death in reoperated patients	6 (2.3 %)	2 (3.5 %)	0.22
Causes of hospital death			
Cardiac	8 (3 %)	3 (5.2 %)	1.0
Septicemia	1 (0.4 %)	0	1.0
Intestinal infarction	1 (0.4 %)	0	1.0
Multi organ failure	3 (1.1 %)	1 (1.7 %)	1.0
Aortic dissection	1(0.4 %)	0	1.0

### Anticoagulation

All patients in Group I were anticoagulated with warfarin sodium from the second postoperative day. All patients in Group II continued the anticoagulation therapy for three months after surgery. The international normalized ratio was used as control and kept between 2.5 and 3.5.

### Follow-up

The mean follow-up was 35 ± 18.5 months (range 13–87 months). The INR, clinical status for any cardiac event were evaluated. Prior discharge all patients underwent echocardiographic examination. The second postoperative echocardiographic control was performed within 1 year after surgery. The mean time-interval between the echocardiographic examination and the operation was 17 ± 5 months. Further follow-up was based on medical records of patients’ medical visits, following echocardiographic examinations (not all of them) and by telephone contacts.

### Echocardiography

Studies were performed with the use of 2.5–3.5 MHz transducer interfaced to SONOS 5500 (Agilent Technologies, Andover, Mass). All patients were monitored with serial echocardiograms; the first study was performed preoperatively, subsequent controls were at discharge and within 1 year postoperatively. Images were stored on tape for late off-line analysis. M-mode, twodimensional, continuous pulsed-wave, and color Doppler were carried out and standard views were used. Measurements of end-systolic diameter (ESD), end-diastolic diameter (EDD), posterior wall thickness (WT), and interventricular septal thickness (IVST) were first made according to the recommendation of the American Society of Echocardiography using a leading edge-to-leading edge convention. Left ventricular ejection fraction (LVEF) was calculated by using the apical four-chamber view and the application of the modified Simpson rule method.

### Doppler measurements and calculations

The maximal instantaneous pressure gradient across the prostheses was estimated by the modified Bernoulli equation; the mean pressure gradient was derived by planimetry of the Doppler envelope. Patient-prosthesis missmatch was defined as those patients with EOA_i_ below 0.75 cm^2^/m^2^ and the severe patient prothesis mismatch was considered when the EOA_i_ was below the 10^th^ percentile (≤0.65 cm^2^/m^2^) [6].

### Definitions

*Body surfacearea (BSA)* is body morphometric analysis indexing weights and heights. Indexed left ventricular mass reduction (R- LVM_i_) is the difference between the preoperative value of the LVM_i_ and postoperative LVM_i_.

### Statistical analysis

Group statistics were expressed as mean ± SD. The generalized Wilcoxon test was performed for the statistical analysis between groups. Fisher’s exact test was used for the non continuous variables. The relationship between preoperative and postoperative variables within the same group was assessed by the McNemar test. The multivariate logistic regression model was employed to determine the predictors for poor early survival. The multivariate Cox proportional regression (Statsoft 6–0) was performed to determine independent predictors. Long-term survival rates were calculated using the Kaplan-Meier method and the long rank test for the comparison between groups. The Spearman linear regression test was employed for analysing the correlation between variables. Significance between data was considered achieved when *p* < 0.05.

## Results

There were 14 (5.3 %) hospital deaths in Group I versus 4(6.8 %) in Group II (*p* = 0.86). The postoperative complications and causes of early deaths are given in Table 3. There were no differences between groups regarding the postoperative complications. The multivariate logistic regression analysis identified the LVEF ≤ 35 %, combined operation, CPB, age, annulus enlargement, reoperation and chronic renal failure as strong predictors for early postoperative death (Table 4).

**Table 4:** Predictors for hospital death and adverse events after aortic valve replacement

	Err.Std.			Err.Std.		
	BETA	di BETA	B	di B	t (265)	*p*-level
*Predictors for early postoperative mortality*						
LVEF ≤ 35 %	−0.31	0.07	−0.21	0.05	−4.52	0.00012
Combined operation	−0.32	0.07	−0.29	0.06	−4.7	0.00005
Annulus enlargement	−0.28	0.069	−0.16	0.04	−4.02	0.0009
CPB	0.12	0.057	0.069	0.032	2.15	0.033
Age	−0.16	0.061	−0.003	0.0012	−2.58	0.011
Reoperation	0.13	0.06	0.08	0.04	2.07	0.039
Chronic renal failure	−0.15	0.06	−0.17	0.065	−2.58	0.011
*Predictors for early postoperative adverse events*						
LVEF ≤ 35 %	−0.27	0.073	−0.33	0.092	−3.63	0.0004
CPB	0.27	0.069	0.008	0.0021	3.87	0.0002
Combined operation	−0.22	0.071	−0.17	0.042	−3.1	0.0031
Annulus enlargement	−0.25	0.086	−0.18	0.062	−3.0	0.0041
Residual gradient	−0.172	0.087	−0.007	0.0033	−1.98	0.049
Chronic renal failure	−016	0.074	−0.155	0.	−2.15	0.033
Reoperation	−0.17	0.073	−0.14	0.06	−2.31	0.022
Endocarditis	0.14	0.07	0.092	0.05	1.99	0.048

*LVEF* left ventricular ejection fraction, *CPB* cardiopulmonary bypass time

All survivors underwent echocardiographic examination at discharge. The mean transprosthesis gradient was 19 ± 9 mmHg in Group I versus 20 ± 5 mmHg in Group II (*p* = 0.2). At six months follow-up the mean NYHA FC class was 1.6 ± 0.5 in Group I and 1.8 ± 0.7 in Group II (*p* = 0.014). However the NYHA FC improved significantly in both groups versus the preoperative values. All survivors underwent transthoracic echocardiography examination at rest within 1 year after surgery (Table 5). Peak pulmonary artery pressure decreased significantly after surgery, (*p* = 0.001 and *p* = 0.002 in Group I and II respectively. The end-diastolic IVS thickness decreased significantly after AVR, (*p* = 0.001 and *p* = 0.012 in Group I and II respectively). The end-diastolic LV posterior wall thickness decreased significantly only in Group I (*p* = 0.035). On the other hand the end-systolic posterior wall thickness decreased significantly in both groups after AVR, (*p* = 0.012 and *p* = 0.026 in Group I and II respectively).

**Table 5:** Postoperative echocardiographic data between groups

Variables	Group I	Group II	*p*-value
Diameter of the ascending aorta (mm)	30 ± 4.2	31.2 ± 7	0.1
Diameter of the left atrium (mm)	45.4 ± 9.4	44.5 ± 7	0.51
End-diastolic right ventricle diameter (mm)	22 ± 8	23 ± 6	0.39
End-diastolic LV diameter (mm)	46.6 ± 6	47 ± 9	0.69
End-systolic LV diameter (mm)	28 ± 6.7	28.6 ± 6	0.55
End-diastolic IVS thickness (mm)	12 ± 1.6^a^	12.2 ± 2.3^a^	0.45
End-systolic IVS thickness (mm)	17.4 ± 5	17.6 ± 4.6	0.79
End-diastolic LV PW thickness (mm)	11.5 ± 1.5^a^	11.3 ± 2.7	0.45
End-systolic LV PW thickness (mm)	16.4 ± 6^a^	16 ± 4^a^	0.86
Shortening Fraction (%)	40.5 ± 9.8	43 ± 11	0.1
End-diastolic LV volume (ml)	85 ± 28.7	91 ± 27	0.16
End-systolic LV volume (ml)	37.5 ± 18.3	40 ± 17	0.36
Indexed end-diastolic LV volume (ml/mq)	50 ± 15	53 ± 17	0.2
Indexed end-systolic LV volume (ml/mq)	21.7 ± 9.7	23.4 ± 11	0.26
Left ventricular ejection fraction (%)	56.4 ± 10.7	55 ± 12	0.4
VmaxE (cm/s)	126 ± 44	122 ± 37	0.54
VmaxA(cm/s)	118.4 ± 29.5	123 ± 33	0.31
Mitral valve regurgitation grade	1.64 ± 0.53^a^	1.5 ± 0.8^a^	0.11
Peak pulmonary artery pressure (mmHg)	43 ± 12.8^a^	40 ± 14^a^	0.13
Left ventricular mass (gm)	199 ± 38^a^	207 ± 61^a^	0.22
Left ventricular mass index (gm/m^2^)	119 ± 22.5^a^	122 ± 22^a^	0.37
Left ventricular mass index reduction (gm/m^2^)	44.1 ± 30	46.4 ± 26	0.60
Peak transvalvular gradient (mmHg)	28.3 ± 11^a^	31 ± 12^a^	0.11
Mean transvalvular gradient (mmHg)	15.7 ± 6.5^a^	17 ± 7^a^	0.19
Aortic valve regurgitation grade	1.1 ± 0.3	1.2 ± 0.9	0.15
Peak trans tricuspid valve gradient	34 ± 11.4	32 ± 13	0.26
Effective orifice area	1.35 ± 0.14	1.33 ± 0.2	0.38
Indexed effective orifice area (cm^2^/m^2^)	0.81 ± 0.12	0.79 ± 0.1	0.26
Indexed orifice area < 0.75 cm^2^/m^2^	110 (43.8 %)	24 (44.4 %)	0.95
Indexed orifice area ≤ 0.65 cm^2^/m^2^	19 (7.5 %)	3 (5.6 %)	0.82
Doppler Velocity Index	0.5 ± 0.15	0.48 ± 0.2	0.41
Mitral valve area (cm^2^)	3.7 ± 6.3	3.5 ± 4.2	0.83

^a^The postoperative value is significantly lower than preoperatively

*LV* left ventricle, *IVS* interventricular septum, *PW* posterior wall

**Fig. 2:**
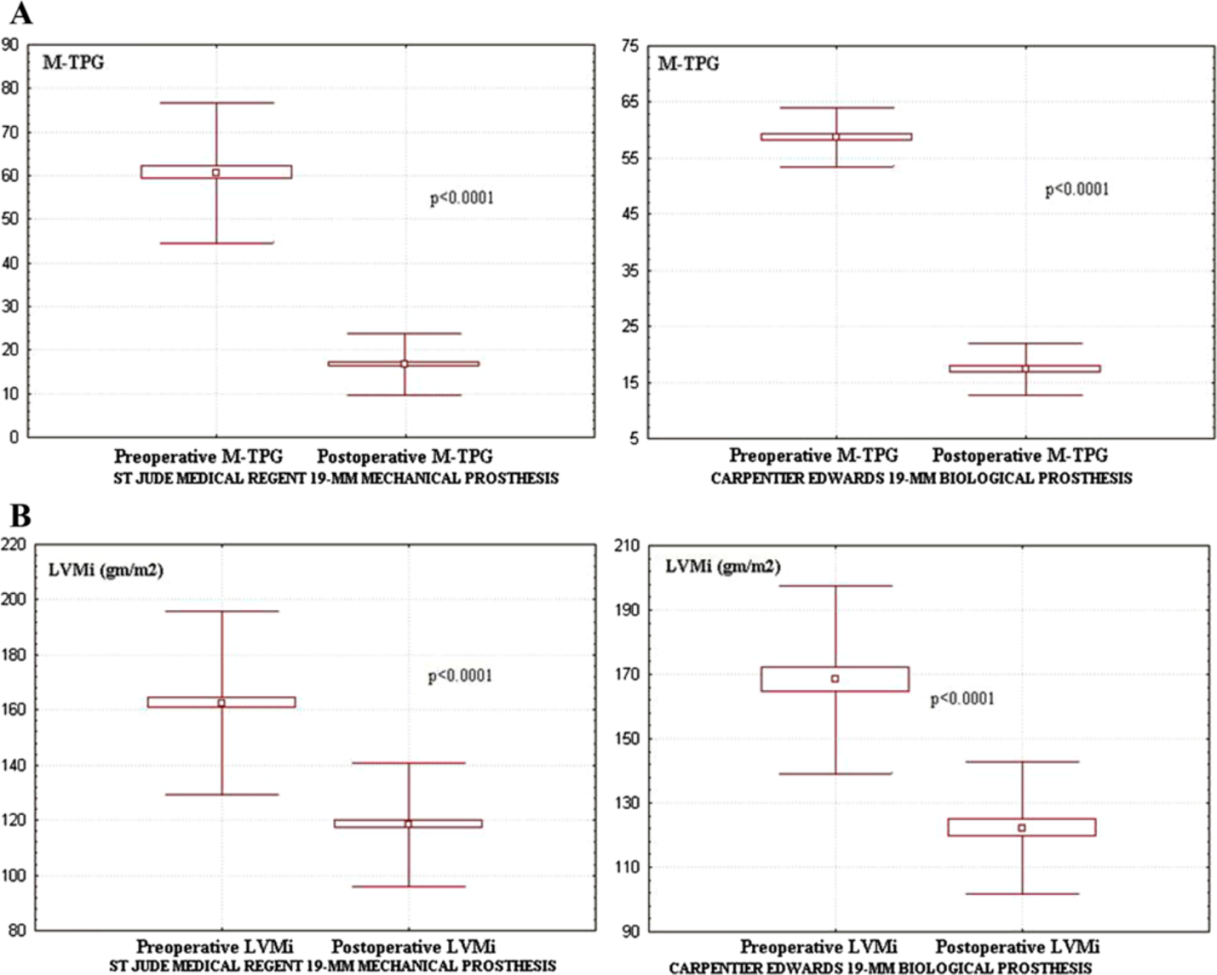
**a**. Postoperative M-TPG versus preoperative value in both groups. **b**. Comparison between the preoperative and postoperative values of the LVMi

The M-TPG was 15.7 ± 6.5 mmHg in Group I versus 17 ± 7 mmHg in Group II (*p* = 0.19), however the M-TPG was significantly lower than preoperatively independently of the employed prosthesis (Fig. 2a). The mean transprosthesis gradient was significantly lower than at discharge in Group I (*p* = 0.003) and Group II (*p* = 0.011). The multivariate regression analysis revealed the annulus enlargement, small EOA_i_, increased LVEDD, postoperative LVM_i_, BSA and older age as strong predictors for higher M-TPG (Table 6).

**Table 6:** Predictors for high mean transprothesis gradients

	Err.Std.			Err.Std.		
	BETA	di BETA	B	di B	t (265)	*p*-level
LVMi (gm/m^2^)	0.4	0.05	7.84	0.97	8.07	0.00000
EOAi (cm^2^/m^2^)	−0.21	0.050210	−0.14	0.033	−4,18	0.00004
LVEDD (mm)	0.097	0.05	−2.33	1.2	−1.95	0.053
Annulus enlargement	0.13	0.054	2.46	1.03	2.4	0.018
Body surface area (m^2^)	0.13	0.052	0.068	0.03	2.35	0.019
Age (years)	0.12	0.055	1.69	0.77	2.18	0.03

*EOAi* aortic indexed effective orifice area, *LVEDD* left ventricular end-diastolic diameter, *LVMi* indexed left ventricular mass

**Fig. 3:**
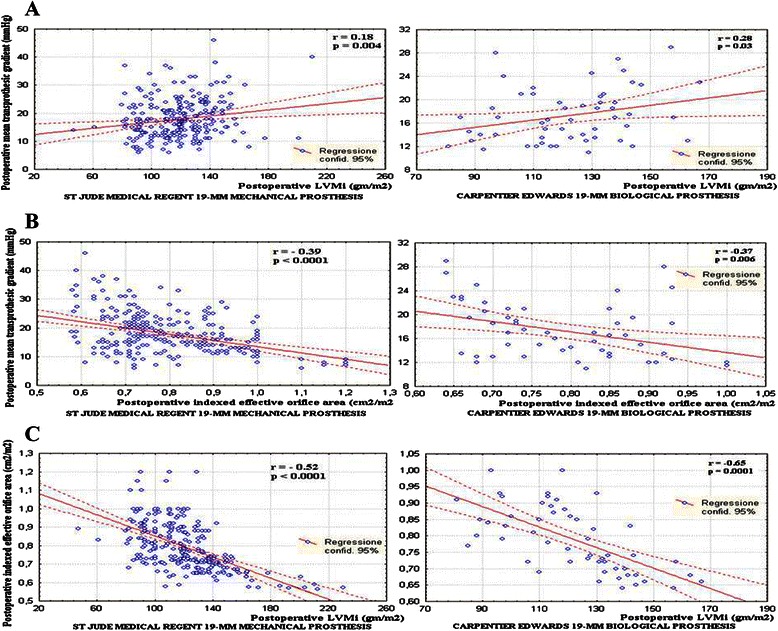
**a**. Correlation between LVMi and M-TPG. Correlation between EOAi and the **b** M-TPG, **c** Postoperative LVMi in both groups

LVM and LVM_i_ were significantly lower than preoperatively independently of the employed prosthesis (Table 5) (Fig. 2b). The R-LVM_i_ was similar between groups (Table 5). The M-TPG had a strong direct correlation with the postoperative LVM_i_ (Fig. 3a) and an indirect correlation was found with the postoperative EOA_i_ (Fig. 3b) independently of the employed SJMR-19 or CE-19. Also the LVM_i_ correlated well with the EOA_i_ independently of the prosthesis type (Fig. 3c).

**Fig. 4:**
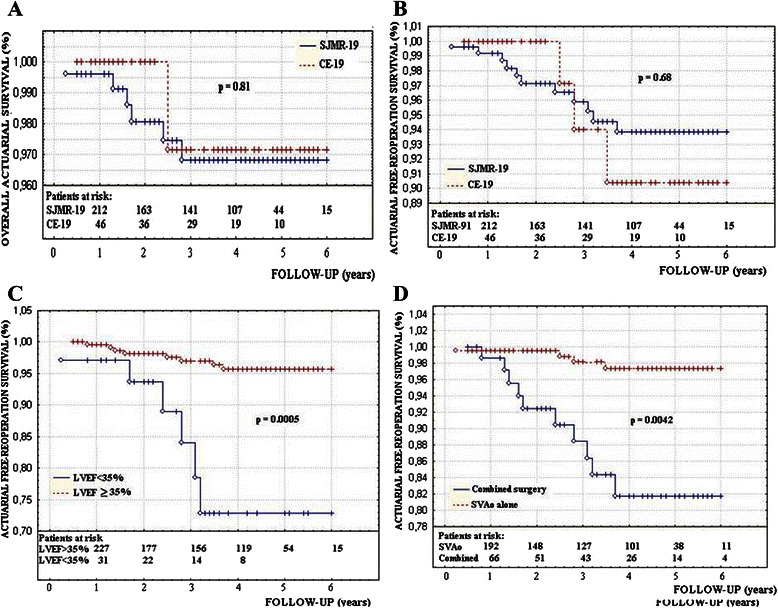
**a** The actuarial survival and **b** cumulative free-reoperation actuarial survival in both groups. The cumulative free-major events actuarial survival in patients with LVEF < 35 % (**c**) and in patients undergoing combined surgery (**d**)

**Fig. 5:**
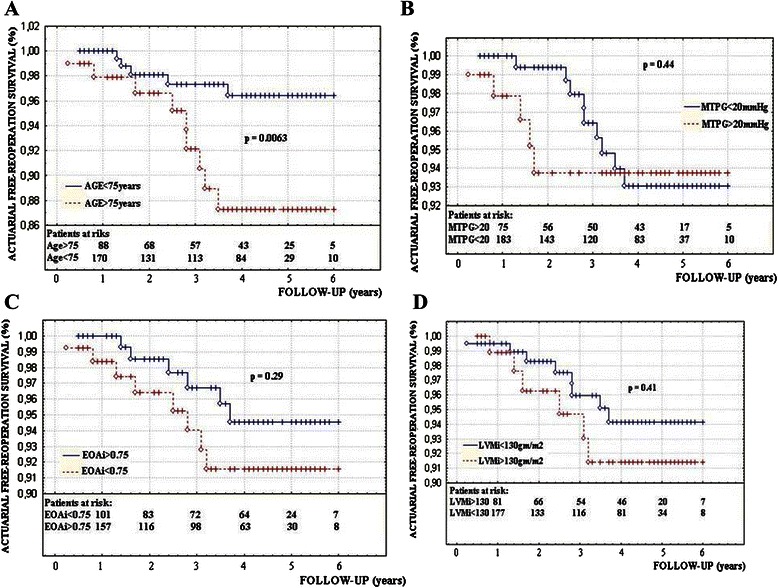
The actuarial free reoperation survival (**a**) in patients older than 75 years, (**b**) in patients with postoperative M-TPG > 20 mmHg, (**c**) in patients with EOAi < 75 and (**d**) in patients with LVMi **≥** 130gm/m2(N)

The actuarial survival and cumulative free-reoperation actuarial survival at 5 years follow-up were 96.7 % and 94.5 % respectively in Group I and 97 % and 91 % in Group II (Fig. 4a and b). There were non significant differences between groups regarding the actuarial survival and cumulative free-reoperation survival. The actuarial free-reoperation survival was significantly lower in patients with LVEF < 35 % (Fig. 4c), undergoing combined surgery (Fig. 4d), in patients older than 75 years (Fig. 5a), although the M-PTG ≥ 20 mmHg, EOA_i_ ≤ 0.75 cm^2^/m^2^ and LVM_i_ ≥ 130gm/m^2^ did not decrease the cumulative actuarial free-reoperation survival (Fig. 5b-d). Other minor complications were identified in seven patients as listed in Table 7.

**Table 7:** Causes of late deaths and adverse events

Variables	Nr (%)	Reoperation
Late deaths		
Cardiac		
Not valve related		
Cardiac arrest	2 (1 in Group II)	No
Valve related		
Endocarditis (reoperation)	2	Yes
Stroke		1
Renal	1	No
Cancer	1	Yes
Unknown	1	Yes
Major adverse events		
Endocarditis	4 (1 in Group II)	2 Yes
Myocardial ischemia	2	Yes
Important paravalvular leak	2 (1 in Group II)	Yes
Aortic dissection	1	Yes
Important mitral valve regurgitation	1 (Group II)	Yes
Minor adverse events		
^a^Renal insufficiency requiring hemodialysis	1	No
Reversible ischemic neurologic deficit	3	No
Moderate paravalvular leak	3	No
Gastric hemorrhage	1	Yes

^a^The same patients requiring reoperation due to endocarditis

We constructed a model including all and adverse events. The Cox model identified age ≥ 75 years, LVEF ≤ 35 %, chronic renal failure, NYHA FC class ≥ 3, reoperation, combined surgery, and annulus enlargement as strong predictors for poor actuarial free-major events survival (Table 8).

**Table 8:** Predictors for overall free-reoperation survival

	Errore			Esponenz	Statist.	
	Beta	Standard	Valore t	beta	di Wald	*p*-value
NYHA FC ≥3	−0.39	0.20	−1.91	0.68	3.66	0.056
Age (years)	−0.67	0.29	−2.3	0.51	5.3	0.022
LVEF ≤ 35 %	0.65	0.25	−2.61	0.53	6.83	0.009
Chronic renal failure	−0.63	0.33	−1.96	0.53	3.83	0.050
Reoperation	0.52	0.22	2.37	1.7	5.63	0.018
Combined surgery	1.02	0.30	3.37	2.78	11.36	0.00075
Annulus enlargement	0.026	0.012	2.13	1.03	4.55	0.033

*LVEF* left ventricular ejection fraction, *NYHA FC* New Yourk Heart Association Functional Class

## Discussion

Management of the small aortic root is a challenge to the surgeon with regard to operative technique and selection of prosthesis. Many techniques have been described [9] to accommodate a larger prosthesis, potentially increasing the risks of injury to the coronary arteries and conduction bundle, and of surgical hemorrhage. On the other hand the stented bioprothesis such as the CE-19 remains an alternative to mechanical prosthesis, homografts, stentless valves and annulus enlargement techniques.

The mechanical prosthesis still remain the most widely use prosthesis for AVR basically due to their proven long-durability, less technical difficulties and costs. The employment of mechanical prosthesis in small aortic annulus is still an issue of controversies in the literature, however, most of surgeons confirm excellent prosthesis hemodynamic in patients undergoing AVR with a small mechanical prosthesis. The SJMR heart valve features a modified outer profile as compared to the standard SJM mechanical heart valve carbon orifice. This modification allows for an increase in the inside lumen area while maintaining the same tissue annulus diameter and sewing cuff diameter as a SJM mechanical heart valve HP valve or a SJM standard mechanical heart valve.

In the present study we report a large series of 265 patients undergoing AVR with a SJMR-19. The early and mid-term clinical and hemodynamic outcome were compared with a group of patients undergoing AVR with a CE-19. The early postoperative outcome in patients with a SJMR-19 were similar to other reported series [14–16]. Similar outcome were found in patients undergoing AVR with a CE-19 [10, 17–21]. The assessment of clinical outcomes relies on long-term assessment for years and decades. However, within the available follow-up of just more than 35 months, there were excellent clinical outcomes among patients independently of the valve type regarding to the improvement of the functional status assessed by New York Heart Association, and there were acceptable adverse events in terms of embolic, bleeding and endocarditis.

The basal transprothesis peak and mean gradients were similar to other series of patients undergoing AVR with SJM standard mechanical prosthesis or SJMHP [5, 22]. Bach et al. [14] reported a significantly lower M-TPG of 9.7 mmHg in 22 patients undergoing AVR with SJMR-19 from a series of 361 patients undergoing AVR with SJMR compared to our series of patients, this probably due to the fact that the mean EOA in this study [14] was 1.6 ± 0.4 cm^2^, higher than our series. In a previous study, the same authors [23] had found a M-TPG of 13.8 mmHg in patients undergoing AVR with SJMR-19. By the other side Gelsomino et al.[15] found a M-TPG of 17 mmHg at 1 year after AVR with SJMR-19 in patients representing a postoperative EOA_i_ = 0.82 cm^2^/m^2^ slightly higher than our series. In another study Sudkamp et al.[16] found a P-TPG of almost 26 mmHg in patients with an EOA of 1.38 cm^2^ undergoing AVR with a SJMR-19. The M-TPG in patients undergoing AVR with a SJMR-19 was lower, eventhough not significant than CE-19. Other studies have demonstrated controversial data regarding the M-TPG in patients with CE-19. The M-TPG for CE-19 range between 12 and 24 mmHg and the EOA between 1.1 cm^2^ and 1.4 cm^2^ [10, 17–21]. In our series we found a 17 mmHg and an EOA of 1.32 cm^2^.

The hemodynamic performance of currently available AV prostheses remains inferior to those of the native AV. All current prosthetic valve designs produce measurable TPG that, potentially, could place persistent additional demands on the LV, and may hinder or delay the regression of LV hypertrophy. This is said to occur more frequently when the size of the implanted prosthesis is limited by the presence of a small aortic annulus, particularly in a patient with a large BSA, when there is a mismatch between prosthesis and patient?! Persistently elevated LVM in these patients, as a result of a small prosthesis with obstructive gradients, may increase the risk of sudden death [8]. The presence of LV hypertrophy in old patients such as the present series with a high incidence of concomitant CABG and at a higher risk of developing severe coronary artery disease postoperatively increases the risk for late deaths [24]. Persistently high postoperative gradients are thought to impair the regression of LVM observed in most patients after surgery [1, 25]. Complete abolition of transvalvular gradients is not possible because of the obstructive effects of the prosthetic valve stents and sewing ring. Although the reduction in LVM_i_ seen in our patients was significant independently of the employed prosthesis type, it should be noted that the average postoperative LVM_i_ remained above the normal range. A similar observation has been made in another study noting a significant regression of LVM with small mechanical valves [26], but observed persistent elevations in mass compared with controls, which were due to persistent septal thickening. There are several possible reasons for incomplete regression of AV stenosis patients may preclude complete regression of LV hypertrophy. It is also possible that although significant regression of LVM had occurred, it may be a continuing process and further reductions of LVM might occur at 2 and 3 years postoperatively. Another possibility is that the AV procedure itself resulted in an increase in LVM that partially offset the benefits of the lower aortic gradient. Thoracotomy itself may increase the LVM [27]. The postoperative LVM and LVM_i_ in our series were significantly lower than preoperatively, indicating that both the implanted SJMR-19 and CE-19 had reduced significantly the pressure gradient across the AV. It may well be argued that during the follow-up, favourable LV remodelling as a result of sustained relief of outflow obstruction has occurred inducing a near normalisation of systolic load following AV replacement associated with a rapid rate of reduction in myocyte hypertrophy and LVM. This was demonstrated even by a significant reduction of the IVS thickness and posterior LV wall hypertrophy in our series independently of the employed prosthesis. These data confirm that both SJMR-19 and CE-19 offers acceptable transprothesis gradients at rest, which are compatible with an almost normal systolic load of the LV. Previous studies, performed in patients receiving 19-mm mechanical prothesis or bioprothesis showed similar results at follow-up [13, 20, 22]. A strong direct correlation between the M-TPG and postoperative LVM_i_ was found both in patients with SJMR-19 and CE-19, as already demonstrated in other series undergoing AVR with larger size mechanical prothesis, however the opinions regarding such correlation remains still controversial [11]. We also found a strong inverse correlation between the M-TPG and EOA_i_ in patients with SJMR-19 and CE-19, similarly to other reported studies [5, 28]. Furthermore, the EOA_i_ inversely correlated with the postoperative LVM_i_. Such data clearly demonstrate the strong effect of the small EOA_i_ on the increased postoperative LVM_i_, independently of the employed prosthesis type.

Patient-prosthesis mismatch has been accepted on the basis of assumptions, indirect evidence, and intuitive reasoning. The postoperative EOA_i_ in our study was similar between SJMR-19 and CE-19 and the mean value was ≤0.85 cm^2^/m^2^ in both groups demonstrating patient-prosthesis missmatch. Patient-prosthesis mismatch has mostly been defined by the presence of abnormal gradients in a setting of decreased EOA_i_. Although most of authors have found the patient-prosthesis missmatch a strong predictor for higher postoperative TPG and late survival the opinions are still controversial [3–8, 29]. For patients with AV stenosis, there is a widely accepted correlation between the reduction of transvalvular gradients and clinical improvement after AVR [1]. Recent studies [2, 4, 5] have demonstrated that patient-prothesis mismatch does not adversely impact on long-term survival, and that valve size may be unimportant. Eventhough we were not able to demostrate in our series the adverse effect of the small EOA_i_ on overall free-reoperation survival, our analysis demonstrated a significant impact of the small EOA_i_ on the postoperative M-TPG. Almost 110 from 251 survivors with SJMR-19 and 24 out of 54 survivors with CE-19 had an EOA_i_ ≤ 0.75 cm^2^/m^2^ which demonstrates a patient-prosthesis mismatch according to definitions given by Rao et al.[8], but very few of them had an EOAi ≤ 0.65 cm^2^/m^2^ according to the new definition given by Goldman et al. [5]. Although the patient-prosthesis mismatch was frequently found in our series independently of the employed prosthesis type, we may hypothetize that the low incidence of severe patient-prothesis mismatch as demonstrated by our data, may have hider and overlaped the real impact of the small EOA_i_ on mid-term free-reoperation survival. Although patient-prosthesis mismatch remains a constant concern in prosthesis’ selection, especially in patients with small aortic root, AVR with the SJMR-19 or CE-19 pericardial bioprosthesis offers excellent midterm results and remains a valuable alternative to aortic root enlargement techniques and to small stentless valves in the elderly.

Other variables such as older age, low LVEF, presence of chronic renal failure, combined surgery associated annulus enlargement and the presence of M-TPG ≥ 20 mmHg were strong predictors for poor overall free-reoperation survival. Sawant et al. [3] in a series of 593 patients undergoing AVR with a SJM 19-mm found the older age as an important predictor for poor survival although the concomitant surgical procedure did not influence long-term survival. He and colleagues [29] reported that the small prosthesis with concomitant CABG was the only negative determinant of long-term survival. In another study [30] the age and heart-related variables were independent risk factors for mortality, thromboembolism, bleeding, serious complications, and all complications joined. Carrier et al. [19] found the older age, male sex, associated CABG and aortoplasty as independent predictors for poor overall free-events survival in a series of 93 patients undergoing AVR with CE-19. The concomitant annulus enlargement was another predictor for poor overall free-events survival in our series confirming other surgical series undergoing annulus enlargement techniques [9] which demonstrated relative success, but at the expense of increased operative risk. In our practice, the indication for annulus enlargement was given in cases when the implantation of a SJMR-19 or CE-19 was not possible in the aortic native annulus. However, the other alternative in such occasions is to implant a smaller valve such as a SJMR-17-mm. During the same study period, we have implanted a SJMR-17 in 17 patients and the initial outcome are encouraging [31, 32]. The annulus enlargement was an independent predictors for poor actuarial overall free-reoperation survival.

The principal concern of the mechanical valve is its thrombogenic potential and the need for anticoagulation. In this series there was no incidence of valve thrombosis. The review by Akins [33] of four common mechanical prosthesis revealed a linearized rate of thromboembolism at 1.6 % patient-years (range, 0.7 to 2.8 %) for the SJM prosthesis. Our findings confirm the durability and minimum thrombogenicity of the SJMR prostheses.

The actuarial survival and overall free-reoperation survival were similar in patients with SJMR-19 versus CE-19. Although almot 40 % of patients underwent concomitant cardiac procedures, in this series the operative mortality and the intermediate survivals were similar with other reported series [3, 13, 14, 29, 32]. The late survival and cumulative free-reoperation survival were strongly related with the older age, combined operation and low LVEF. Eventhough this study demonstrate similar hemodynamic performance between the SJMR-19 and CE-19, the long-term durability of the CE-19 is much more lower than the mechanical prosthesis due to proven structural valve failure. For this reason, in our practice, we have indicated the employement of the CE-19 only in elderly patients.

### Study limitations

1) One of the major limitations of the continuous–wave Doppler is the possibility of overestimation of valvular flow velocity and pressure gradients and underestimation of the valve area by the continuity equation, as a consequence of pressure-recovery phenomenon. 2) Another limitation is the older age of patients undergoing AVR with a SJMR-19 or CE-19. These patients have reduced daily physical activities and as consequence might be “false” assymptomatic. 3) The patients in this study were not homogeneous and were included patients undergoing double valve replacement or coronary artery bypass grafting, although the loading conditions on a prosthetic valve should be similar in all patient subgroups.

## Conclusion

We may conclude that the SJMR-19 and CE-19 offers acceptable postoperative clinical and hemodynamic outcome in patients with small aortic annulus. The LV hypertrophy and transvalvular gradients are reduced significantly, independently of the employed prosthesis type. Our data demonstrates that small valve size (19 mm) of St Jude Regent and Carpentier Edwards are associated with acceptable mid-term free-reoperation survival. The hemodynamic results of CE-19 mm are similar to SJMR-19 in old patients.
